# Split-Dose Versus Standard Single-Dose Bowel Preparation Regimens for Colonoscopy: A Systematic Review and Meta-Analysis

**DOI:** 10.7759/cureus.107530

**Published:** 2026-04-22

**Authors:** Yanqing Sun, Airong Xu, Dayan Zhang, Lin Ba

**Affiliations:** 1 Gastroenterology, Nursing, Binzhou People's Hospital, Binzhou, CHN; 2 Endoscopy Center, Nursing, Binzhou People's Hospital, Binzhou, CHN; 3 Oncology, Nursing, Binzhou People's Hospital, Binzhou, CHN; 4 Anesthesiology, Binzhou People's Hospital, Binzhou, CHN

**Keywords:** bowel preparation, colonoscopy, meta-analysis, polyethylene glycol, single-dose, split-dose, systematic review

## Abstract

Adequate bowel preparation is essential for colonoscopy. While split-dose regimens may improve cleansing by shortening the interval to the procedure, the comparative efficacy of split-dose regimens versus single-dose regimens remains under investigation. This meta-analysis evaluated whether split-dose bowel preparation is superior to single-dose regimens for adequate cleansing and polyp detection.

We systematically searched PubMed, EMBASE, Web of Science, and Cochrane CENTRAL through February 20, 2026, for randomized controlled trials (RCTs) comparing split-dose versus single-dose preparation in adults undergoing colonoscopy. The primary outcome was adequate bowel preparation rate, and the secondary outcome was polyp detection rate. A random-effects model with the Hartung-Knapp-Sidik-Jonkman (HKSJ) method was used to pool risk ratios (RRs).

Five RCTs (n=1,004) were included, of which only two studies were eligible for primary outcome synthesis. Split-dose preparation was associated with a numerically higher adequate cleansing rate (RR=1.24; 95% HKSJ CI: 0.67-2.31; P=0.32; I²=67%). Polyp detection showed a similar non-significant trend (RR=1.42; 95% CI: 0.14-14.67; P=0.68; I²=65%). A supplementary meta-analysis of three studies reporting continuous scores demonstrated significantly better cleansing with split-dose regimens (standardized mean difference = 0.85; 95% CI: 0.22-1.48; P=0.03).

A split-dose preparation significantly improved cleansing quality on continuous scales, but the pooled analysis of adequate preparation rates showed a non-significant trend. However, the evidence was limited by heterogeneous outcome reporting.

## Introduction and background

Colonoscopy remains the gold standard for colorectal cancer screening and prevention, and its diagnostic and therapeutic efficacy is critically dependent on the quality of bowel preparation [1,2]. Inadequate bowel cleansing can lead to missed lesions, prolonged procedure time, increased patient discomfort, and the need for early repeat examinations, imposing significant burdens on both patients and healthcare systems [3,4].

Split-dose bowel preparation, defined as administering at least half of the laxative volume 4-6 hours before colonoscopy, has emerged as a strategy to optimize cleansing quality [5]. The underlying rationale is pharmacological: shortening the interval between the final dose and the procedure minimizes the accumulation of small intestinal secretions and prevents the dilution of the cleansing agent, thereby improving mucosal visualization [6,7]. Despite its theoretical advantages and endorsement by major gastroenterology societies [8], concerns regarding patient compliance, sleep disruption, and logistical challenges related to early-morning dosing have tempered its universal adoption [9].

While previous systematic reviews have suggested potential benefits of split-dose regimens, their conclusions have been limited by heterogeneity in bowel preparation formulations, dosing schedules, and outcome measurement tools [10,11]. Furthermore, many existing meta-analyses have pooled studies using different cleansing scales (e.g., Boston Bowel Preparation Scale (BBPS), Ottawa Scale, Aronchick Scale) without adequately accounting for the clinical implications of these differences [12-15]. The relationship between improved cleansing quality and clinically meaningful endpoints such as polyp detection also remains incompletely characterized.

Therefore, this systematic review and meta-analysis aimed to synthesize available randomized controlled trial (RCT) evidence with the following focused hypothesis: Does split-dose bowel preparation achieve superior rates of adequate bowel cleansing and polyp detection compared with standard single-dose regimens in adult patients undergoing elective colonoscopy? We sought to provide a rigorous, contemporary synthesis while transparently addressing the limitations imposed by heterogeneous outcome reporting.

## Review

Methods

Study Design and Population, Intervention, Comparator, Outcomes (PICO) Framework

This systematic review was structured according to the PICO framework to ensure clarity and reproducibility. The population (P) consisted of adult patients (aged ≥18 years) undergoing elective colonoscopy for screening, surveillance, or diagnostic indications. The intervention (I) was split-dose bowel preparation, defined as administration of at least 50% of the total laxative volume on the day of the procedure (typically within 3-6 hours before colonoscopy). The comparator (C) was a standard single-dose bowel preparation, defined as the administration of the entire laxative volume on the day prior to the procedure. The primary outcome (O) was the rate of adequate bowel preparation, using each study's predefined criteria based on validated scales (e.g., BBPS total score ≥6 or segmental score ≥2, Ottawa Scale score below a specified threshold, or investigator-defined "adequate" cleansing). The secondary outcome was polyp detection rate, defined as the proportion of patients with at least one polyp identified during colonoscopy.

Eligibility Criteria

Inclusion criteria were: (1) RCT design; (2) direct comparison of split-dose versus single-dose bowel preparation regimens; (3) adult population undergoing elective colonoscopy; and (4) reporting of at least one prespecified outcome (bowel preparation quality or polyp detection).

Exclusion criteria were: (1) non-randomized study designs (e.g., observational cohorts, case-control studies); (2) studies involving pediatric populations or emergency procedures; (3) studies not reporting discrete outcome data for each study group; and (4) studies in which the bowel preparation regimen was part of a multifactorial intervention without isolated comparison.

Search Strategy

This systematic review was conducted and reported in accordance with the Preferred Reporting Items for Systematic Reviews and Meta-Analyses (PRISMA) 2020 statement [16]. We systematically searched PubMed, EMBASE, Web of Science, and the Cochrane Central Register of Controlled Trials (CENTRAL) from database inception to February 20, 2026. The search strategy combined terms for the population, intervention, comparator, and study design using Boolean operators. The core search logic for PubMed was as follows:

(("Colonoscopy"[Mesh] OR "colonoscopy"[tiab]) AND ("Split-Dose Bowel Preparation"[tiab] OR "split-dose prep"[tiab] OR "split bowel preparation"[tiab]) AND ("Single-Dose Bowel Preparation"[tiab] OR "single-dose prep"[tiab] OR "single bowel preparation"[tiab])) AND ((randomized controlled trial [pt] OR controlled clinical trial [pt] OR randomized [tiab] OR placebo [tiab] OR drug therapy [sh] OR randomly [tiab] OR trial [tiab] OR groups [tiab]) NOT (animals [mh] NOT humans [mh]))

No language restrictions were applied. The reference lists of all included studies and relevant systematic reviews were manually screened for additional eligible trials. Although the search was updated through February 20, 2026, only a limited number of head-to-head randomized controlled trials directly comparing split-dose versus standard single-dose regimens met the prespecified eligibility criteria.

Study Selection

**Figure 1:**
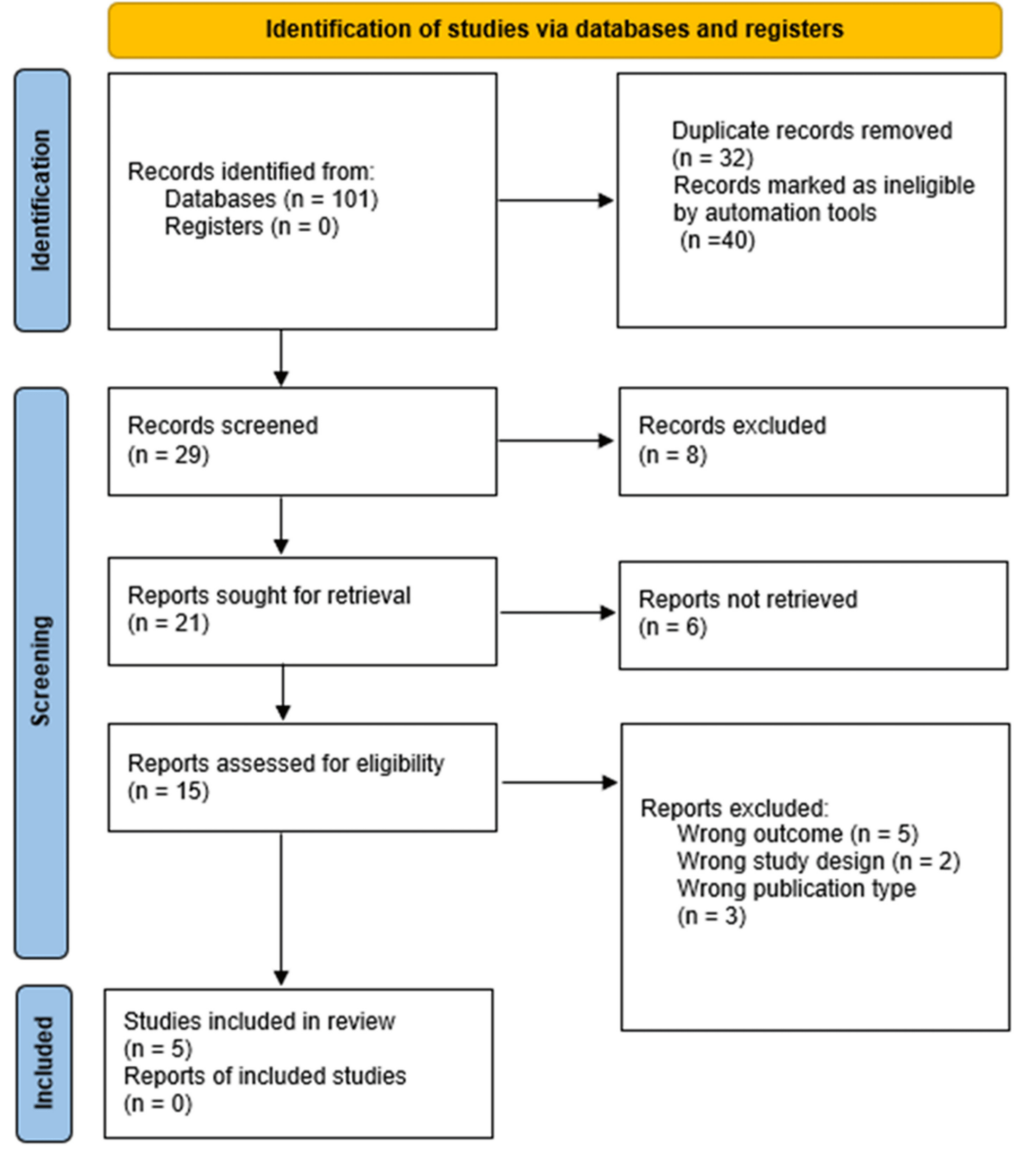
PRISMA 2020 flow diagram of study selection This figure illustrates the flow of studies through the systematic review process, including identification, screening, eligibility, and inclusion stages. Identification: Records identified from PubMed (n = 41), EMBASE (n = 34), Web of Science (n = 20), and Cochrane CENTRAL (n = 6). Screening: Duplicates were removed using EndNote (Clarivate Plc, Philadelphia, PA, USA) and Rayyan (Rayyan Systems, Inc., Cambridge, MA, USA). PRISMA: Preferred Reporting Items for Systematic Reviews and Meta-Analyses.

Two independent reviewers screened titles and abstracts and subsequently full-text articles against the eligibility criteria. Disagreements were resolved through consensus or consultation with a third reviewer. The study selection process is detailed in the PRISMA flow diagram (Figure 1).

Data Extraction and Outcome Definitions

Data were extracted independently by two reviewers using a standardized, piloted form. Extracted variables included: first author, publication year, country, patient population characteristics, bowel preparation formulation and dosage, details of split-dose and single-dose regimens, timing of the final dose relative to colonoscopy, definitions of adequate bowel preparation, and numbers of events for bowel preparation adequacy and polyp detection in each group.

For the primary outcome of "adequate bowel preparation," we accepted each study's definition as reported. To facilitate meta-analysis, we mapped these definitions onto a binary outcome reflecting clinically acceptable mucosal visualization. For studies using the BBPS, "adequate" was defined as a score of ≥2 in all colonic segments. For studies using a global 5-point scale (e.g., excellent, good, adequate, fair, poor), we classified "excellent," "good," and "adequate" as the adequate group. The potential impact of this definitional heterogeneity on the pooled estimate was considered in the interpretation of the results.

Secondary outcomes were polyp detection rate and, where reported, adenoma detection rate (ADR) and advanced adenoma detection rate. We prioritized ADR as a more clinically relevant quality indicator for colonoscopy. However, due to inconsistent reporting of ADR in the primary studies, only the polyp detection rate was amenable to quantitative synthesis; ADR data were summarized narratively where available.

Risk of Bias Assessment

The methodological quality of each included RCT was assessed independently by two reviewers using the revised Cochrane Risk of Bias tool for randomized trials (RoB 2) [17]. This tool evaluates bias across five domains: the randomization process, deviations from intended interventions, missing outcome data, measurement of the outcome, and selection of the reported result. Each domain was judged as "low risk of bias," "some concerns," or "high risk of bias," with an overall risk of bias determined for each study.

Statistical Analysis

All analyses were performed using R software (version 4.3.2) with the meta and metafor packages [18]. For dichotomous outcomes (adequate bowel preparation, polyp detection), pooled risk ratios (RRs) with 95% confidence intervals (CIs) were calculated. A random-effects model was applied a priori for all pooled analyses. Due to the small number of studies included in the meta-analysis, we used the Hartung-Knapp-Sidik-Jonkman (HKSJ) method with the Restricted Maximum Likelihood (REML) estimator for between-study variance (τ²) to provide more conservative 95% confidence intervals (CIs) compared to the standard DerSimonian-Laird method [19,20]. For the primary analysis, we also calculated the 95% prediction interval for the pooled effect size, which estimates the range within which the true effect in a future study is expected to lie [21].

Statistical heterogeneity was quantified using the I² statistic, with values of 25%, 50%, and 75% representing low, moderate, and high heterogeneity, respectively [22]. For continuous outcomes (bowel cleansing scores reported on different scales, such as the Ottawa Scale and BBPS), we calculated the standardized mean difference (SMD) with 95% HKSJ CI to pool effects across studies. A positive SMD was defined to consistently favor the split-dose regimen.

Given the limited number of studies eligible for meta-analysis (<10), formal assessment of publication bias using funnel plots and Egger's test was not performed, as these methods have low statistical power with small study numbers [23]. All statistical tests were two-sided, with P < 0.05 considered statistically significant.

Protocol and Registration

This systematic review was not prospectively registered in PROSPERO or another publicly accessible registry. However, the review protocol, including the PICO framework, eligibility criteria, outcome definitions, and analysis plan, was finalized prior to the commencement of data extraction and screening. All analyses presented were pre-specified; no post-hoc deviations from the initial plan were made.

Results

Study Selection and Characteristics

The study selection process is detailed in the PRISMA flow diagram (Figure 1). A total of five RCTs published between 2007 and 2021, involving 1,004 patients undergoing colonoscopy, met the predefined inclusion criteria [24-28].

The included studies varied in geographic origin (Korea, United States, Mexico, Oman), patient populations (general outpatients, low-risk outpatients, inpatients), and bowel preparation formulations (polyethylene glycol (PEG) 4L, PEG 2L with ascorbic acid, low-volume PEG-based solutions, and MiraLAX/Gatorade combinations). Split-dose regimens consistently involved administration of half the preparation on the day before colonoscopy and the remaining half on the procedure day, while single-dose regimens involved full preparation on the day prior. Key characteristics of all included studies are summarized in Table 1.

**Table 1:** Characteristics of the included studies for the meta-analysis BBPS: Boston Bowel Preparation Scale; PEG: polyethylene glycol; PEG-ELS: polyethylene glycol-electrolyte lavage solution

Study	Country/Population	Preparation	Split-Dose Regimen	Single-Dose Regimen	Primary Outcome Scale/Definition
Park et al., 2007 [24]	Korea (screening population)	PEG 4L	3L evening prior + 1L procedure day	4L evening prior	Ottawa Scale (segmental and total scores)
Samarasena et al.. 2012 [25]	USA (low-risk outpatients)	Golytely or MiraLAX/Gatorade	Split-dose (details not specified) vs single-dose (both 2×)	Single-dose (details not specified)	BBPS and Ottawa Scale
Téllez-Ávila et al., 2014 [26]	Mexico (inpatients)	PEG (Nulytely)	2L evening prior + 2L procedure day	4L evening prior	BBPS (segmental satisfaction rates)
Horton et al., 2016 [27]	USA (outpatients)	2L PEG-ELS + ascorbic acid	Half evening prior + half 4 hours pre-procedure	2L evening prior	Endoscopist-recorded preparation quality (Excellent/Good/Adequate/Fair/Poor)
Al Alawi et al., 2021 [28]	Oman (outpatients)	PEG 4L (Nulytely)	2L evening prior + 2L morning of procedure	4L afternoon to midnight prior	BBPS (0–1 inadequate, 2–3 adequate)

Risk of Bias Assessment

**Figure 2:**
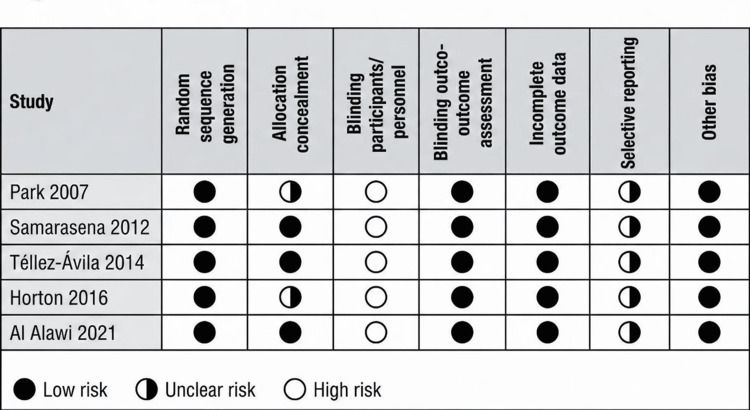
Risk of bias summary This figure (traffic light plot) summarizes the risk of bias (RoB) assessments in each domain of the Cochrane RoB 2 tool across all five included studies [24-28]. The domains are: D1, randomization process; D2, deviations from intended interventions; D3, missing outcome data; D4, measurement of the outcome; and D5, selection of the reported result.

**Figure 3:**
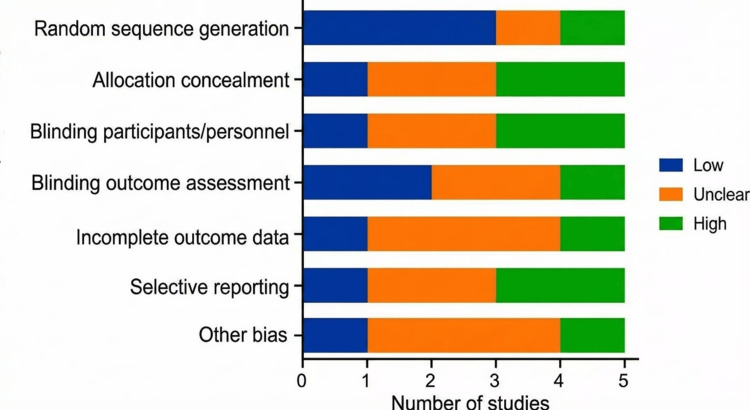
Risk of bias results This graph (weighted summary plot) presents the proportion of studies judged to have low risk, some concerns, or high risk of bias for each domain across all five included studies [24-28].

**Figure 4:**
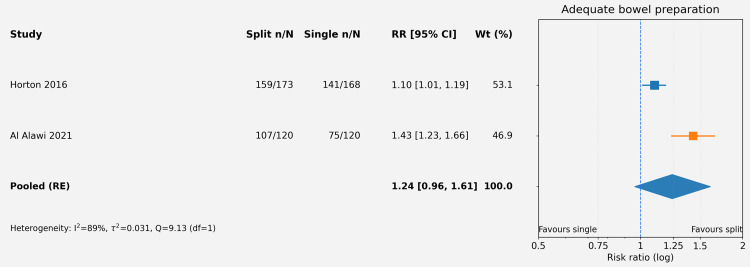
Forest plot of pooled risk ratio for adequate bowel preparation comparing split-dose versus single-dose regimens The forest plot displays the study-specific and pooled RRs with 95% CIs for the primary outcome (adequate bowel preparation) comparing split-dose with single-dose regimens. The pooled estimate (diamond) was derived from a random-effects model using the Hartung-Knapp-Sidik-Jonkman (HKSJ) method with the Restricted Maximum Likelihood (REML) estimator. The studies included correspond to references [27,28]. RR: risk ratio; CI: confidence interval

**Figure 5:**
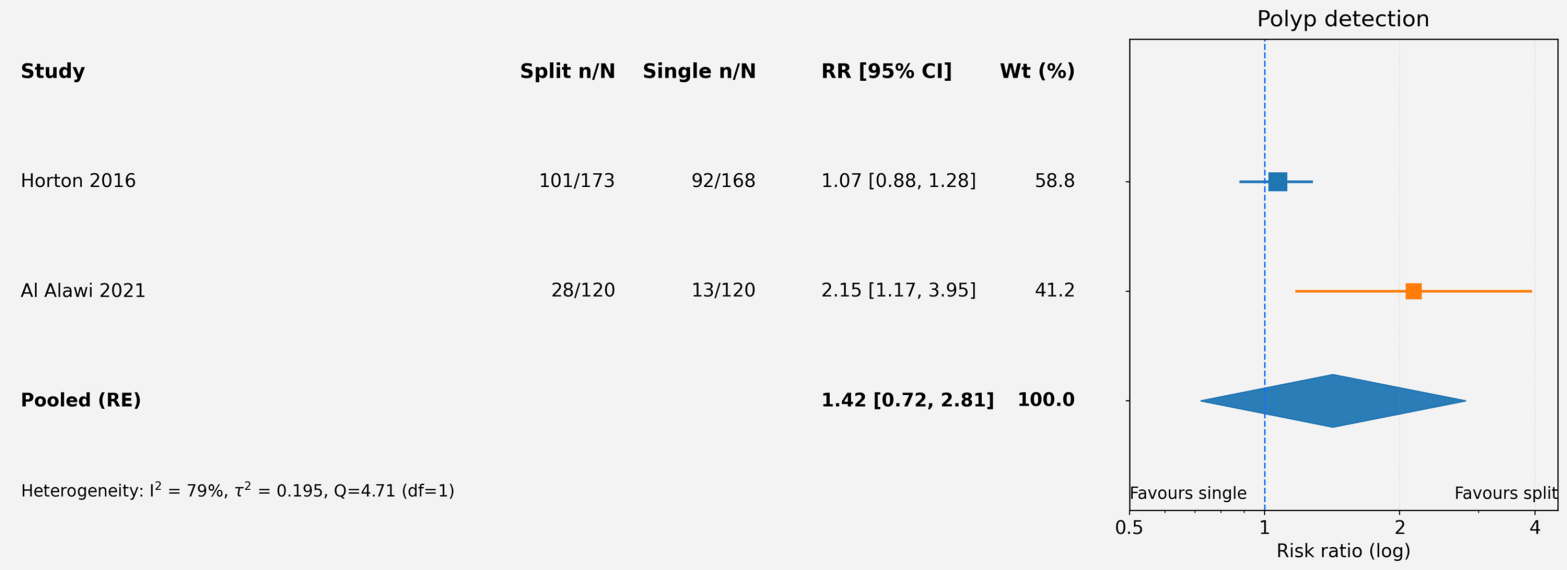
Forest plot of pooled risk ratio for polyp detection comparing split-dose versus single-dose regimens The forest plot displays the study-specific and pooled RRs with 95% CIs for the secondary outcome (polyp detection) comparing split-dose with single-dose regimens. The pooled estimate (diamond) was derived from a random-effects model using the Hartung-Knapp-Sidik-Jonkman (HKSJ) method with the Restricted Maximum Likelihood (REML) estimator. The studies included correspond to references [27,28]. RR: risk ratio; CI: confidence interval

The methodological quality of the five included RCTs was assessed using the Cochrane RoB 2 tool [17]. Overall, the risk of bias was judged as low in three studies (60%), with some concerns identified in two studies, primarily related to blinding of personnel and outcome assessment (deviations from intended interventions and measurement of the outcome domains). The randomization process (Domain 1) and selection of reported results (Domain 5) were generally robust across studies. Figures 2, 3 summarize the domain-specific and overall risk of bias assessments. Figures 4, 5 summarize the pooled risk ratio for adequate bowel preparation and polyp detection in split-dose versus single-dose regimens.

Synthesis of Continuous Cleansing Scores

To further leverage the available data, we performed a quantitative synthesis of continuous cleansing scores. Three studies reported bowel cleanliness using validated continuous scales: Park et al. [24] and Samarasena et al. [25] used the Ottawa Scale (lower scores indicate better cleansing), while Téllez-Ávila et al. [26] used the Boston Bowel Preparation Scale (BBPS; higher scores indicate better cleansing). Due to the opposing direction of these scales, we converted all effects to a standardized mean difference (SMD), where a positive SMD consistently favored the split-dose regimen.

A meta-analysis of these three studies (n = 380 patients) using a random-effects model (HKSJ-REML) demonstrated that split-dose preparation was associated with significantly better cleansing quality compared to single-dose regimens (SMD = 0.85, 95% HKSJ CI: 0.22-1.48; P = 0.03). Substantial heterogeneity was observed (I² = 75%, τ² = 0.24). The 95% prediction interval ranged from -1.01 to 2.71. This finding supports the primary analysis and suggests that the benefit of split-dose regimens is more clearly demonstrated when using continuous, rather than dichotomized, outcome measures. Details of the data extraction for this analysis are provided in Supplementary Table 2.

Qualitative Synthesis of Non-Pooled Studies

Among the three studies not included in the quantitative meta-analysis of dichotomous outcomes, all reported findings generally consistent with a benefit of split-dose regimens. Park et al. (2007) reported significantly better Ottawa Scale scores (indicating superior cleansing) with split-dose compared with single-dose PEG 4L preparation (mean total Ottawa score 3.6 vs. 5.2, P < 0.001) [24]. Samarasena et al. (2012) found that split-dose MiraLAX/Gatorade preparation achieved higher rates of "excellent/good" preparation compared with single-dose regimens (97% vs. 86%, P < 0.05) [25]. Téllez-Ávila et al. (2014) reported that split-dose 4L PEG achieved significantly higher rates of adequate preparation (BBPS ≥2 in all segments) compared with single-dose 4L PEG (90% vs. 62%, P < 0.05) [26].

Discussion

This systematic review and meta-analysis of five RCTs comparing split-dose versus single-dose bowel preparation for colonoscopy found a consistent directional trend favoring split-dose regimens for improving cleansing quality, although the pooled estimate for adequate bowel preparation did not reach statistical significance. The point estimate (RR = 1.24) suggests a potential 24% relative increase in the likelihood of achieving adequate cleansing with split-dose administration, which is clinically meaningful if confirmed in larger studies. However, the confidence interval crossing unity and the substantial statistical heterogeneity mandate cautious interpretation.

The observed trend aligns with the pharmacological rationale underlying split-dose regimens. By shortening the interval between the final laxative dose and colonoscopy, split-dose administration minimizes the time available for small intestinal secretions to accumulate and dilute the colonic effluent, thereby optimizing mucosal visualization [6,7]. This mechanism is particularly relevant for morning colonoscopy schedules, where a single-dose regimen administered entirely the previous day results in a prolonged interval (12-18 hours) during which the cecum and right colon may become re-soiled with bile-stained fluid [29].

The non-significant finding for polyp detection (RR = 1.42) reflects the anticipated relationship between improved visualization and lesion identification, but the wide confidence interval and substantial heterogeneity preclude definitive conclusions. It is mechanistically plausible that superior cleansing quality should translate into higher polyp detection, particularly for smaller or flat lesions that may be obscured by residual debris [30]. However, the two studies contributing to this analysis had markedly different baseline polyp detection rates (54.8-58.4% in Horton et al. vs. 10.8-23.3% in Al Alawi et al.), likely reflecting differences in patient populations, screening versus diagnostic indications, and endoscopic techniques, which contributed to the statistical heterogeneity [27,28]. Accordingly, the polyp detection analysis should be interpreted as exploratory and hypothesis-generating rather than confirmatory.

A central finding of our analysis is the substantial limitation imposed by heterogeneous outcome reporting. Among five eligible RCTs, only two reported bowel preparation quality as a dichotomous outcome using definitions that could be harmonized for meta-analysis. The remaining studies reported continuous scale scores (mean Ottawa or BBPS) without providing dichotomized event data, or used ordinal categories that could not be reliably mapped to a standardized "adequate" threshold. This variability in outcome measurement represents a major barrier to evidence synthesis and has been identified as a persistent challenge in bowel preparation research [12,31]. The Consolidated Standards of Reporting Trials (CONSORT) extension for non-pharmacological trials and core outcome set initiatives may help address this issue by promoting standardized reporting [32].

Limitations

Several limitations must be acknowledged when interpreting these findings. First, despite including five RCTs, the limited number of studies eligible for quantitative synthesis (n = 2) precluded meaningful exploration of sources of heterogeneity through meta-regression or subgroup analyses. For the same reason, we did not perform formal small-study effect or publication bias analyses, as funnel plots and related statistical tests are unreliable and potentially misleading when very few studies are available [23].

Second, the two pooled studies used different definitions of adequate bowel preparation: Horton et al. used a 5-point global assessment scale, while Al Alawi et al. used the BBPS with a threshold of segmental score ≥2 [27,28]. While both approaches were mapped to a binary "adequate" outcome in our analysis, they may capture different aspects of cleansing quality, and this definitional heterogeneity likely contributed to the observed statistical heterogeneity (I² = 67%). This limitation underscores the need for standardized outcome definitions in bowel preparation research.

Third, this review was not prospectively registered in PROSPERO. Although the protocol was finalized prior to data extraction and all analyses were pre-specified, the absence of registration introduces a potential risk of reporting bias, and readers should consider this when assessing the evidence.

Fourth, the included studies spanned a 15-year publication period (2007-2021), during which endoscopic technology, bowel preparation formulations, and quality benchmarks evolved, potentially introducing temporal heterogeneity.

Fifth, while we focused on the clinically meaningful endpoints of adequate cleansing and polyp detection, we were unable to perform a quantitative synthesis of adenoma detection rate (ADR), a more robust quality indicator for colonoscopy, due to inconsistent reporting in the primary studies. ADR data were summarized narratively where available, but this represents an important gap in the evidence base. Patient-centered outcomes such as tolerability, willingness to repeat preparation, sleep disturbance, and adverse events (nausea, bloating, abdominal pain) were also not systematically assessed due to inconsistent reporting. These factors are critical for shared decision-making, as concerns about sleep disruption and early-morning travel remain barriers to split-dose adoption [9].

Sixth, the continuous outcomes meta-analysis, while demonstrating a significant benefit for split-dose regimens, was limited by the heterogeneity in the scales used (Ottawa vs. BBPS) and the assumption that a positive SMD uniformly favors split-dose regimens. The wide prediction interval (-1.01 to 2.71) indicates substantial uncertainty in the true effect size.

Implications for Practice and Future Research

Patient education and logistical support are essential for the successful implementation of split-dose regimens. Clear instructions regarding the timing of the second dose, expected bowel movement patterns, and strategies to minimize sleep disruption can improve adherence [33]. For patients with significant concerns about early-morning dosing or those with limited mobility, shared decision-making should weigh the potential improvement in cleansing quality against individual preferences and practical constraints.

Future research should prioritize several key areas. First, the adoption of standardized outcome definitions and reporting formats is urgently needed to facilitate evidence synthesis. Core outcome sets for bowel preparation research, including both efficacy endpoints (adequate cleansing rates using validated scales with prespecified thresholds) and patient-centered outcomes (tolerability, acceptability, sleep interference), would enable more robust meta-analyses [34]. Second, large pragmatic RCTs comparing split-dose regimens with novel low-volume preparations in diverse patient populations (elderly, patients with comorbidities, those with previous inadequate preparation) would clarify effect modification and inform personalized prescribing. Third, studies should systematically collect and report data on polyp detection, adenoma detection rate, and advanced adenoma detection as clinically meaningful downstream outcomes. Fourth, comparative effectiveness research using standardized protocols and blinded outcome assessment would strengthen the evidence base and reduce the risk of bias identified in some included studies.

## Conclusions

This systematic review and meta-analysis found that split-dose bowel preparation was associated with significantly better cleansing quality when analyzed using continuous scale scores. However, the pooled analysis of dichotomous adequate preparation rates, based on only two trials, showed a non-significant trend favoring split-dose regimens. The effect on polyp detection should be interpreted cautiously because of limited data, wide confidence intervals, and substantial heterogeneity.

Overall, the direction of effect is broadly consistent with current guideline preferences for split-dose regimens. However, the certainty of the quantitative pooled estimates remains limited. The major barrier to firmer conclusions is the heterogeneity in outcome definitions and reporting across trials. Future research should prioritize standardized outcome reporting, including both clinically interpretable adequacy thresholds and lesion-detection endpoints, to enable more robust evidence synthesis and better inform practice.

## Appendices

**Table 2:** Data extraction for meta-analysis BBPS: Boston Bowel Preparation Scale; PEG: Polyethylene Glycol; Go-Si: Golytely single-dosed; Go-Sp: Golytely split-dosed; Mlax-Si: MiraLAX in Gatorade single-dosed; Mlax-Sp: MiraLAX/Gatorade split-dosed

Study	Scale used	Direction of scale	Single-dose group (n)	Split-dose group (n)	Extracted outcome data reported in source	Notes for synthesis
Park et al., 2007 [24]	Ottawa Scale	Lower score = better cleansing	152	151	The study used the Ottawa scale and reported better bowel preparation with split-dose PEG than with single-dose PEG. In the manuscript extraction, mean total Ottawa score was summarized as 5.2 for single-dose vs 3.6 for split-dose. The article page provided also shows lower Ottawa scores in split-dose compliance subgroups than in single-dose subgroups, supporting the same direction of effect.	Effect direction coded so that improvement favored split-dose.
Samarasena et al., 2012 [25]	Ottawa Scale; BBPS	Ottawa Scale: lower = better; BBPS: higher = better	Go-Si: 57; Mlax-Si: 60	Go-Sp: 51; Mlax-Sp: 54	Mean Ottawa scores: Go-Si 6.77, Go-Sp 4.12, Mlax-Si 6.25, Mlax-Sp 4.80. Mean BBPS scores: Go-Si 6.07, Go-Sp 8.33, Mlax-Si 6.62, Mlax-Sp 8.01. The study found significantly better cleansing with split-dose regimens than whole-dose regimens.	For the supplementary synthesis in the review, Ottawa-scale results were used because the review text classifies this study under continuous cleansing scales using Ottawa. If only one comparison was entered per study, clarify in methods whether Golytely, MiraLAX/Gatorade, or pooled split-vs-single arms were used.
Téllez-Ávila et al., 2014 [26]	BBPS	Higher score = better cleansing	60	61	The study compared single-dose 4 L PEG vs split-dose 2 L + 2 L PEG vs same-day 2 L PEG. For the review question, the relevant comparison is group 1 single-dose (n=60) vs group 2 split-dose (n=61). Reported data in the article are segment-specific satisfactory cleansing rates rather than a clearly stated total BBPS mean±SD: right colon 53% vs 84%, transverse colon 69% vs 85%, left colon 67.7% vs 78.3% (single-dose vs split-dose).	Because this paper reports BBPS results as segment-level satisfactory rates, the exact transformation used for the supplementary quantitative synthesis should match the review team’s extraction worksheet/R script. If the review used a derived continuous summary, that derived value should be inserted here from the original analysis file.
